# Use of Menopausal Hormone Therapy and Bioidentical Hormone Therapy in Australian Women 50 to 69 Years of Age: Results from a National, Cross-Sectional Study

**DOI:** 10.1371/journal.pone.0146494

**Published:** 2016-03-23

**Authors:** Louiza S. Velentzis, Emily Banks, Freddy Sitas, Usha Salagame, Eng Hooi Tan, Karen Canfell

**Affiliations:** 1 Cancer Research Division, Cancer Council NSW, Sydney, NSW, Australia; 2 National Centre for Epidemiology and Population Health, Research School of Population Health, Australian National University, Canberra, ACT, Australia; 3 School of Public Health, University of Sydney, Sydney, NSW, Australia; 4 Saw Swee Hock School of Public Health, National University of Singapore, Singapore, Singapore; 5 Lowy Cancer Research Centre, University of New South Wales, Sydney, NSW, Australia; University of North Carolina School of Medicine, UNITED STATES

## Abstract

Menopausal Hormone Therapy (MHT) use in Australia fell by 55% from 2001 to 2005, following the release of large-scale findings on its risks and benefits. Comprehensive national data, including information on overall prevalence of MHT use as well as information on duration of use in Australia have not been reported since the 2004–5 National Health Survey, when 11% of women aged 45+ years were estimated to be current MHT users. No national data are available on prevalence of use of “bioidentical” hormone therapy (BHT). The objective of this study was to determine recent prevalence of MHT and BHT use. A cross-sectional, national, age-stratified, population survey was conducted in 2013. Eligible women, aged 50–69 years, resident in Australia were randomly sampled in 5-year age groups from the Medicare enrolment database (Australia’s universal health scheme). The response rate was 22% based on return of completed questionnaires, and analyses were restricted to 4,389 women within the specified age range. The estimated population-weighted prevalence of current use of MHT was 13% (95%CI 12–14), which was broadly similar to the previously reported national figures in 2004–5, suggesting that the use of MHT in Australia has largely stabilised over the past decade. A total of 39% and 20% of current-users with an intact uterus reported use of oestrogen-progestagen MHT and oestrogen-only MHT, respectively, whereas 77% of hysterectomised current-users used oestrogen-only MHT. Almost three-quarters of current-users [population-weighted prevalence 9% (95%CI 8–10)] had used MHT for ≥5 years. In regard to BHT, estimated population-weighted prevalence of ever use was 6% (95%CI 6–7) and 2% (95%CI 2–3) for current use. The population-weighted prevalence of MHT and BHT combined, in current users in their fifties and sixties was 15% (95%CI 14–16). These data provide a recent national “snapshot” of Australian women’s use of both conventional MHT and of BHT.

## Introduction

Menopausal hormone therapy (MHT) is an effective treatment for vasomotor symptoms associated with the menopause [1].However, MHT use for relief of these symptoms is recommended for the shortest duration possible and should not be used for the prevention of chronic disease [2–4]. In 2002, the US Women’s Health Initiative (WHI) randomised controlled trial of combined oestrogen-progestagen MHT was stopped early due to evidence of excess risk of serious disease including invasive breast cancer [5]. Publication of the WHI trial results prompted rapid falls in use of MHT. Analysis of trends in annual MHT prescribing in Australia demonstrated a 40% drop in MHT use from 2001 to 2003 among women aged 50 years and older [6]; and a 55% drop from 2001–2005 [7]. Consistent with these findings, a National Health Survey conducted in 2004–5 found that the prevalence of self-reported current use of MHT in women over 45 years was 11% [8], a fall from 21% which was reported in 2001 for women ≥50 years of age [9]. Since these initial falls in MHT use were documented, there has been limited information [10–11] on more recent trends in national prevalence of MHT use in Australia with no data on duration or type of MHT. This is important because independent quantitative synthesis of the evidence has found an increased risk for breast cancer in MHT users, with these risks being higher in women using combined oestrogen-progestagen MHT; relative risks increase with increasing duration of use (e.g. compared to never-users, summary relative risks for breast cancer for current combined MHT users for 5 and 10 years were estimated at 1.6 and 2.2, respectively) but return to baseline soon after women cease use [4]. A recent case-control analysis of risk factors for breast cancer in Australian women (controlling for other confounding factors) has also demonstrated broadly comparable findings [12]. Recently, the number of MHT attributable cancers in Australia was estimated using overall and long term MHT exposure based on the 2004–5 National Health Survey, and it was estimated that 453 breast cancers were attributable to combined MHT in 2010 [13].

Bioidentical hormones are defined as chemical substances that are identical in molecular structure to human hormones [14]. ‘Bioidentical hormone therapy’ (BHT) is a term generally used to describe plant-derived, hormone formulations (most commonly combinations of progesterone and 17β oestradiol, oestrone or oestriol) specifically compounded for an individual. In the context of safety concerns about MHT (especially for long term duration use),BHT has been promoted as a more natural and safer alternative for relieving menopausal symptoms, but it has been noted that the safety and efficacy of compounded hormone products has not been extensively evaluated in large scale randomised trials [15]. A 2008 South Australian survey of women reported current and past use of ‘bioidentical imported hormonal mixtures compounded by chemists in troches or creams’ by 6.9% of women ≥ 50 years old [16], but no national data on prevalence of BHT use in Australia are available.

Therefore the aim of the current study was to provide recent prevalence estimates for MHT and BHT use by 5-year age groups, in Australian women 50–69 years of age. We did this via a national, population based survey, the LADY (Learning how Australians Deal with menopause sYmptoms) study.

## Materials and Methods

The LADY study is a cross-sectional, national study for females 50–69 years of age. Eligible women (i.e. women aged 50–69 years) resident in Australia were sampled from the Medicare enrolment database of the Department of Human Services (formerly Medicare Australia), which is Australia’s national healthcare insurance scheme. From the end of January 2013 until the end of February the Department of Human Services sent women an invitation letter, an information sheet and consent form, and a 37-item questionnaire. The questionnaire assessed demographic and lifestyle characteristics, medical information, past and current use of MHT, BHT, and other menopausal therapies. Forms were returned to the study coordinating centre at Cancer Council NSW. Women who returned completed consent forms and questionnaires were considered to be participants and were allocated study numbers. Data were de-identified.

Information collected about use of MHT included ever use, current use, total duration of use, the name of the most recently used proprietary preparation and duration of its use. Questions on MHT use and type were based on previously validated questions [17], although some questions were updated to take into account the range of currently available formulations. Based on their constituents, MHT preparations were grouped as: oestrogen-only; oestrogen-progestagen combinations; progestagen-only; tibolone; “other” [including hormonal preparations for local use and use of more than one type of MHT (systemic, local or combination of both)]; and “unknown”. BHT was defined in the questionnaire as “natural/bioidentical hormones custom-made for you by a compounding pharmacist”. Information collected about use of BHT included ever use, current use, total duration of use, reasons for use and whether MHT was used prior to BHT. The questionnaire also assessed whether participants had undergone a hysterectomy and if so their age at hysterectyomy.

An age-stratified, random sampling method was used, whereby women were randomly sampled within 5-year age groups (50–54, 55–59, 60–64 and 65–69 years). Sample size estimates were based on the relative proportions of MHT prevalence by 5-year age group reported in the National Health Survey [9] and a 2% prevalence of current use of BHT in women aged 50–54 was used for sample size estimates as a conservative assumption. This gave a minimum precision of +/-1.25% for age-specific estimates by 5-year age group based on the assumed prevalence of BHT. A sample size of 3,425 women was estimated to be required and a total of 20,000 women from all Australian States were invited to participate. The response rate was calculated from returned questionnaires and returned opt-out forms. Prevalence and 95% confidence intervals for MHT and BHT use were estimated using the Wald method. Women with missing data were excluded from relevant prevalence estimates (missing data was 4% or less) but for completeness all participants in each age category were reported. To obtain population-weighted estimates for women aged 50–69 years the age-specific prevalence estimates were weighted to 2012 Australian midyear population estimates using the population proportion in each age group [18].

Ethical approval for the LADY study was obtained from the Cancer Council NSW Human Research Ethics Committee on the 19th December 2011, project reference number 256.

## Results

A total of 4,428 women (22.1%) returned a consent form and a completed questionnaire and 1,656 (8.3%) returned an opt-out form, resulting in an overall response rate of 22.1% for completed forms and 30.4% for all respondents. Thirty-nine participants did not provide enough details to calculate their age or were above 69 years of age and therefore the analysis was restricted to the remaining 4,389 participants who returned completed questionnaires. Demographic characteristics of participants are presented in Table 1 and characteristics of the female Australian population from the 2011 Census are also presented for comparison. In general, participants in the study were broadly comparable with the Australian female population of the same age for levels of education, marital status and employment status. For example, the proportion of LADY study participants with year 12 (final year of high school) or equivalent qualifications for age groups 50–54, 55–59, 60–64 and 65–69 years were 12% (95%CI 10–14), 10% (95%CI 8–12), 11% (95%CI 9–13) and 11% (95%CI 10–13), respectively and the respective proportions for women in the general Australian population were 13%, 12%, 12%, 11% (although it should be noted that the proportion of women in their late sixties with no school certificate was lower in the study than observed in the population). Study participants were somewhat more likely to be born in Australia (the questionnaire was provided only in the English language) and slightly less likely to reside in major cities.

**Table 1:** Summary of key demographic characteristics of LADY study participants compared to female population data from the 2011 Census.

		Australian population				LADY participants			
		Proportion in %				% (95%CI)*			
Indicator	Classes	Age Groups (years)				Age Groups (years)			
		50–54	55–59	60–64	65–69	50–54 (n = 962)	55–59 (n = 824)	60–64 (n = 928)	65–69 (n = 1675)
Country of	Australia	64	64	62	62	74 (70.8–76.3)	76 (72.9–78.8)	75 (72.0–77.6)	72 (69.5–73.9)
Birth	Overseas	31	31	33	32	26 (23.6–29.1)	24 (21.1–26.9)	25 (22.3–27.9)	28 (26.0–30.3)
Remoteness	Major city	68	67	66	65	60 (56.8–63.0)	58 (54.4–61.1)	57 (54.0–60.4)	56 (53.7–58.5)
Of	Inner regional	20	21	22	23	24 (21.2–26.6)	24 (21.6–27.5)	25 (22.2–27.8)	26 (24.0–28.3)
Residence	Outer regional + remote +	12	12	12	12	15 (12.7–17.3)	15 (12.7–17.6)	15 (12.4–16.9)	16 (14.1–17.6)
	very remote								
	No school certificate	9	12	18	24	8 (6.5–10.0)	11 (10.9–13.1)	14 (12.0–16.5)	12 (10.8–13.9)
Highest	Year 10 or equivalent	26	28	27	31	19 (16.8–21.8)	22 (19.4–25.0)	25 (22.4–28.0)	31 (28.5–33.0)
Qualifications	Year 12 or equivalent	13	12	12	11	12 (10.1–14.3)	10 (7.6–11.6)	11 (8.6–12.5)	11 (9.8–12.8)
	Certificate/Diploma	22	23	17	17	27 (24.7–30.4)	27 (23.8–19.8)	20 (17.4–22.5)	20 (17.8–21.6)
	University degree or higher	21	19	15	12	33 (29.6–35.6)	30 (27.0–33.2)	30 (26.8–32.7)	22 (20.0–24.0)
	Married	63	65	65	63	70 (66.9–72.7)	65 (61.9–68.4)	68 (65.1–71.1)	68 (66.3–70.7)
Marital	Never Married	11	8	5	4	16 (13.8–18.5)	17 (14.9–20.1)	10 (7.9–11.7)	3 (2.4–4.1)
Status	Separated	5	4	4	3	4 (2.6–5.1)	4 (2.8–5.5)	3 (2.1–4.4)	2 (1.6–3.0)
	Divorced	18	18	17	15	9 (7.1–10.7)	10 (7.5–11.5)	13 (11.2–15.6)	12 (9.9–13.0)
	Widowed	3	5	9	15	1 (0.6–2.1)	4 (2.5–5.1)	6 (4.0–7.0)	12 (9.9–13.0)
Employment	In labour force	74	63	41	17	84 (81.9–86.5)	70 (67.3–73.5)	45 (41.0–47.4)	17 (15.4–19.1)
Status	Not in labour force	22	33	54	77	14 (12.0–16.4)	29 (25.3–31.5)	54 (51.2–57.6)	82 (79.7–83.4)

*Missing values are excluded from proportions reported but were less than 3%

For all women aged 50–69 years, the population-weighted prevalence of ever use of MHT was 37% (95%CI 36–39). When considering ever use in specific age groups, 20%, 33%, 47%, and 57% of women in the age groups 50–54, 55–59, 60–64 and 65–69 years, respectively, reported being ever users of MHT (Table 2). For all women aged 50–69 years, the population-weighted prevalence of current use was 13% (95%CI 12–14). When considering current use in specific age groups, the prevalence was 11% in the 50–54 year age group, 14% in the 55–59 and 60–64 year age groups, and 11% in women aged 65–69 years (Table 2). In terms of duration of use, overall, for women aged 50–69 years, the estimated proportion using MHT for <5 years was 27% of current users [population-weighted prevalence 4% (95%CI 4–5)] and the estimated proportion using MHT for ≥5 years was 73% of current users [population-weighted prevalence 9% (95%CI 8–10)]. As expected the proportion of long term current users increased with age; 83% and 93% of current users aged 60–64 and 65–69 years, respectively, had used MHT for ≥ 5 years.

**Table 2:** Prevalence, type and duration of MHT in current, past and ever users in 5-year age groups and population-weighted proportions for participants 50–69 years of age in the LADY study.

AGE GROUPS (Years)										
	50–54		55–59		60–64		65–69		TOTAL 50–69^1^	
	N	%(95%CI)^2^	N	%(95%CI)^2^	N	%(95%CI)^2^	N	%(95%CI)^2^	N	%(95%CI)^2^,^3^#
**Total Participants**	962		824		928		1675		4389	
**Never used MHT**	763	79.3 (76.8–81.9)	550	66.7 (63.5–70.0)	484	52.2 (48.9–55.4)	712	42.6 (40.2–45.0)	2509	62.2 (60.7–63.6)
**CURRENT USERS**										
**Any current MHT**	110	11.4 (9.4–13.4)	119	14.4 (12.0–16.8)	134	14.4 (12.2–16.7)	184	11.0 (9.5–12.5)	547	12.9 (11.8–13.9)
**MHT by TYPE**										
Oestrogen (systemic)	37	3.8 (2.6–5.1)	47	5.7 (4.1–7.3)	56	6.0. (4.5–7.6)	100	6.0 (4.8–7.1)	240	5.3 (4.6–6.0)
Oestrogen+Progestagen	34	3.5 (2.4–4.7)	33	4.0 (2.7–5.3)	28	3.0 (1.9–4.1)	31	1.9 (1.2–2.5)	126	3.2 (2.6–3.8)
Progestagen (systemic)	4	0.4 (0–0.4)	1	0.1 (-0.1–0.4)	1	0.1 (-0.1–0.3)	1	0.1 (-0.1–0.2)	7	0.2 (0–0.3)
Tibolone	12	1.2 (0.5–1.9)	13	1.6 (0.7–2.4)	22	2.4 (1.4–3.3)	14	0.8 (0.4–1.3)	61	1.5 (1.1–1.9
Other^4^	22	2.3 (1.3–3.2)	23	2.8 (1.7–3.91)	25	2.7 (1.7–3.7)	36	2.2 (1.5–2.8)	106	2.5 (2.0–3.0)
Do not recall	1	0.1 (-0.1–0.3)	2	0.2 (-0.1–0.6)	2	0.2 (-0.1–0.5)	2	0.1(0–0.3)	7	0.2 (0.0–0.3)
**MHT DURATION**										
<5 year	71	7.4 (5.7–9.0)	40	4.9 (3.4–6.3)	22	2.4 (1.4–3.3)	12	0.7 (0.3–1.1)	145	4.2 (3.5–4.9)
≥5 years	38	4.0 (2.7–5.2)	78	9.5 (7.5–11.5)	111	12.0 (9.9–14.0)	172	10.3 (8.8–11.7)	399	8.6 (7.7–9.5)
**PAST USERS**										
**Any past MHT**	79	8.2 (6.5–9.9)	147	17.8 (15.2–20.5)	301	32.4 (29.4–35.4)	763	45.6 (43.2–47.9)	1290	24.0 (22.8–25.2)
**MHT by TYPE**										
Oestrogen (systemic)	24	2.5 (1.5–3.5)	41	5.0 (3.5–6.5)	84	9.1 (7.2–10.9)	255	15.2 (13.5–16.9)	404	7.3 (6.5–8.0)
Oestrogen+Progestagen	9	0.9 (0.3–1.5)	23	2.8 (1.7–3.9)	16	1.7 (0.9–2.6)	38	2.3 (1.6–3.0)	86	1.9 (1.5–2.3)
Progestagen (systemic)	8	0.8 (0.3–1.4)	7	0.8 (0.2–1.5)	15	1.6 (0.8–2.4)	31	1.9 (1.2–2.5)	61	1.2 (0.9–1.6)
Tibolone	9	0.9 (0.3–1.5)	6	0.7 (0.1–1.3)	30	3.2 (2.1–4.4)	24	1.4 (0.9–2.0)	69	1.5 (1.2–1.9)
Other^4^	6	0.6 (0.1–1.1)	9	1.1 (0.4–1.8)	18	1.9 (1.1–2.8)	67	4.0 (3.1–4.9)	100	1.7 (1.4–2.1)
Do not recall	23	2.4 (1.4–3.4)	61	7.4 (5.6–9.2)	138	14.9 (12.6–17.2)	348	20.8 (18.8–22.7)	570	10.4 (9.5–11.2)
**MHT DURATION**										
<5 years	68	7.1 (5.4–8.7)	102	12.4 (10.1–14.6)	158	17.0 (14.6–19.4)	293	17.5 (15.7–19.3)	621	12.9 (11.9–14.0)
≥5 years	10	1.0 (4.0–1.7)	44	5.3 (3.8–6.9)	139	15.0 (12.7–17.3)	457	27.3 (25.2–29.4)	649	10.7 (9.9–11.6)
**EVER USERS**^5^										
**Any MHT**	195	20.3 (17.7–22.8)	273	33.1 (29.9–36.3)	437	47.1 (43.9–50.3)	955	57.1 (54.7–59.5)	1860	37.4 (36.0–38.9)
**MHT by TYPE**										
Oestrogen (systemic)	62	6.4 (4.9–8.0)	91	11.0 (8.9–13.2)	140	15.1 (12.8–17.4)	358	21.4 (19.4–23.4)	651	12.7 (11.7–13.7)
Oestrogen+Progestagen	43	4.5 (3.2–5.8)	57	6.9 (5.2–8.7)	44	4.7 (3.4–6.1)	69	4.1 (3.2–5.1)	213	5.1 (4.4–5.8)
Progestagen (systemic)	12	1.2 (0.5–1.9)	8	1.0 (0.3–1.6)	17	1.8 (1.0–2.7)	33	2.0 (1.3–2.6)	70	1.5 (1.1–1.8)
Tibolone	22	2.3 (1.3–3.2)	18	2.2 (1.2–3.2)	52	5.6 (4.1–7.1)	38	2.3 (1.6–3.0)	130	3.1 (2.5–3.6)
Other^4^	28	2.9 (1.8–4.0)	33	4.0 (2.7–5.3)	44	4.7 (3.4–6.1)	105	6.3 (5.1–7.4)	210	4.3 (3.7–4.9)
Do not recall	21	2.2 (1.3–3.1)	57	6.9 (5.2–8.7)	131	14.1 (11.9–16.4)	318	19.0 (17.1–20.9)	527	9.6 (8.8–10.5)
**MHT DURATION**										
<5 year	142	14.8 (12.5–17.0)	143	17.4 (14.8–19.9)	180	19.4 (16.9–21.9)	307	18.4 (16.5–20.2)	772	17.3 (16.1–18.5)
≥5 years	48	5.0 (3.6–6.4)	122	14.8 (12.4–17.2)	250	26.9 (24.1–29.8)	629	37.6 (35.3–39.9)	1049	19.3 (18.2–20.5)

^1^ Age-standardised prevalence estimates provided for women aged 50–69 years.

^2^ Missing values have been excluded from the proportions reported but were 4% or less.

^3^ Note: 3 participants stated having a levonorgestrel IUD (progesterone-releasing IUD, generally used for contraceptive purposes) but reported taking no other MHT preparation. These women have not been included under MHT user

^4^Other: MHT for local use and combination of the different MHT types

^5^Twenty three women did not specify whether they had stopped using MHT and were classified as ever users.

The predominant type of MHT that current users reported using was oestrogen-only, followed by oestrogen-progestagen therapy, other (defined as use of more than one type of MHT and/or oestrogen-only therapy for localised use), tiboloneand progestagen-only MHT; their relative proportions were 44%, 23%, 19%, 11% and 1%, respectively (missing/do not recall: 2%). In current users, the relative proportion of oestrogen-only users was 34% in women aged 50–54 years (4% of all participants in this age group) and 54% in those aged 65–69 years (6% of all participants in this age group). Conversely, use of combined oestrogen-progestagen preparations was 31% in current users in the 50–54 year group (4% of all participants in this age group) and 17% in the 65–69 year group (2% of participants in this age group).

**Fig 1:**
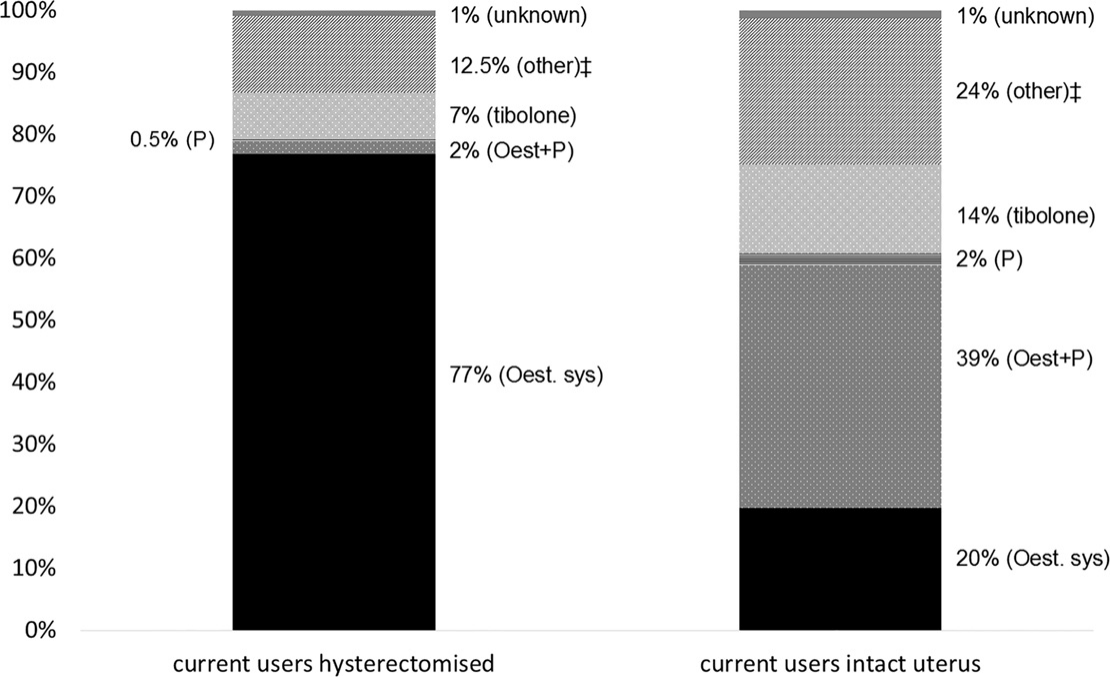
Types of MHT used by current users, in hysterectomised women and in women with an intact uterus. * Oest: oestrogen; P: progestagen; sys: systemic. * Missing values have been excluded from the proportions but are 4% or less. ‡ MHT for local use and combination of different MHT types.

Of all current users, 57% reported that they had an intact uterus and 43% reported having a hysterectomy. In women with an intact uterus, the predominant MHT types in current users were combination MHT (39%), oestrogen-only MHT (20%), and tibolone (14%). Use of oestrogen-only MHT reported by current users with an intact uterus was 15%, 16%, 19%, 26% of women aged 50–54, 55–59, 60–64, 65–69 years, respectively. In hysterectomised current users, oestrogen-only MHT (77%) and tibolone (7%) were the predominant types reported with only 2% of women reporting use of combination MHT (Fig 1). In the LADY study cohort, the population-weighted estimate of women with hysterectomy was 26% (95%CI 25–27) and the age-specific estimates of hysterectomy prevalence were 19%, 23%, 31%, 35% for women 50–54, 55–59, 60–64 and 65–69 years, respectively.

Overall, the population-weighted estimate of use of BHT in women aged 50–69 years was 6% (95%CI 6–7), and for current use of BHT was 2% (95%CI 2–3) (Table 3). In current users, based on small numbers, prevalence of BHT use was 3% in women 50–54 years, and 1% in women aged 65–69 years. A total of 5% of current users (n = 12) reported that they had switched from MHT to BHT. Based on reported current use of MHT and BHT combined, the overall population-weighted prevalence for women 50–69 years was estimated to be 15% (95% 14–16).

**Table 3:** Prevalence of BHT in current and ever users by 5 year age groups and weighted prevalence in participants 50–69 years of age in the LADY study.

	AGE GROUPS (years)				
	50–54	55–59	60–64	65–69	50–69
	N %(95%CI)*	N %(95%CI)*	N %(95%CI)*	N %(95%CI)*	N %(95%CI)*‡
**Participants**	962	824	928	1675	4389
**Never used BHT**	877 91.4 (89.6–93.1)	758 92.0 (90.1–93.8)	858 92.5 (90.8–94.2)	1555 92.8 (91.6–94.1)	4048 92.1 (91.2–92.9)
**CURRENT USERS**					
**BHT**	31 3.2 (2.1–4.3)	20 2.4 (1.4–3.5)	14 1.5 (0.7–2.3)	22 1.3 (0.8–1.9)	87 2.2 (1.7–2.7)
**PAST USERS**					
**BHT**	36 3.8 (2.5–5.0)	34 4.1 (2.8–5.5)	39 4.2 (2.9–5.5)	56 3.3 (2.5–4.2)	165 3.9 (3.3–4.5)
			**EVER USERS**^1^		
**BHT**	67 7 (5.4–8.6)	56 6.8 (5.1–8.5)	55 5.9 (4.4–7.4)	80 4.8 (3.8–5.8)	258 6.2 (5.5–7.0)

*Missing values were excluded from proportions reported but were less than 3%.

‡ Age-standardised prevalence estimates (population-weighted prevalence) provided for women aged 50–69 years.

^1^Six women did not report whether they had stopped using BHT and were classified as ever users only.

## Discussion

**Fig 2:**
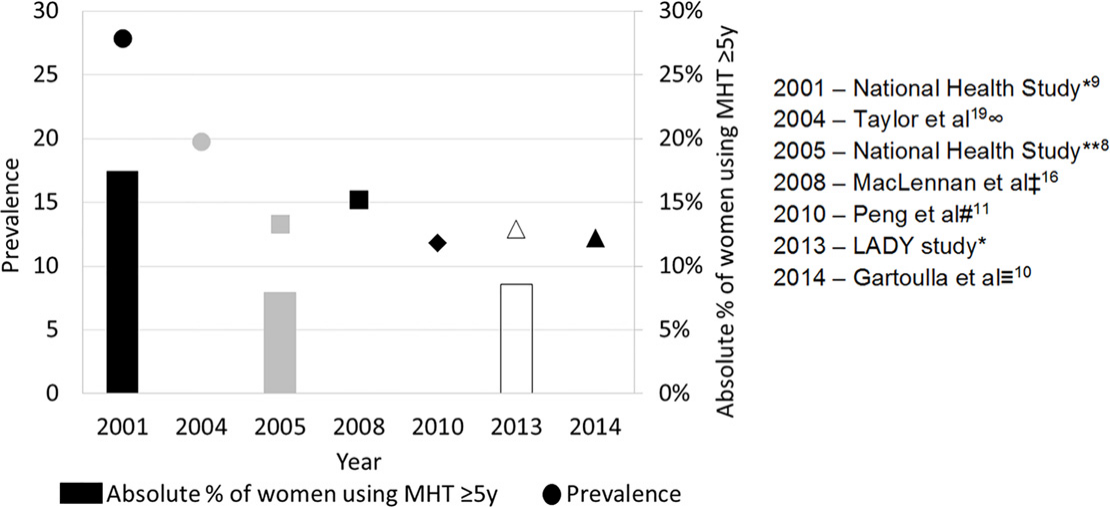
Summary of findings for prevalence of current MHT use and absolute percentage of MHT users who reported use for ≥5 years, over the period 2001 to 2014, in Australian studies (Bars represent absolute percentage of women using MHT for 5 years or more and the dot points represent the overall prevalence of current use reported by each corresponding study). * Data presented for women aged 50–69 years. ∞ Data presented for women aged 50 and over from Taylor et al [19]. Duration of MHT use not reported. **Data presented for women aged 45–64 years. Further subdivision by age was not possible. Prevalence of MHT for women aged 45+ (including women aged 65 and over) was 11%. ‡ Data presented for women aged 50–69 years, calculated from reported use in women aged 50–59 and 60–69 years and from population estimates in 2008. # Data presented for women aged 59–64 years. ≡ Data presented for women aged 40–65 years.

The current study is one of the few sources of national data on prevalence of MHT use in Australia in the last decade. To our knowledge, this is the first national study to evaluate the prevalence of MHT use according to both the duration of use and the type of MHT, and considering whether or not women reported having had a hysterectomy. Taking into account differences in the age range of women surveyed, our finding for the overall prevalence of current MHT use in women aged 50–69 years of 13% is broadly similar to the 11% prevalence in women aged ≥45years reported in the National Health Survey of 2004–5 [8]. Our findings for overall prevalence of use of MHT are also comparable to other previous Australian studies. Data on prevalence of MHT was reported by a study investigating the use of complementary and alternative medicines for menopausal symptoms [10], which found that 12.18% of surveyed women aged 40–65 years in 2014 used MHT. Similarly, in another 2010 study collecting data on women’s health [11] the prevalence of MHT reported for women aged 59–64 years was 12%. In South Australia, current use of MHT in women aged 50–69 years was reported as 15.2% in 2008 [16]. Although differences in the age ranges and survey methods must be taken into account, taken together, these findings suggest that the overall prevalence of current use of MHT in women in their fifties and sixties has stabilised at around 12–15% over the past decade, following an initial rapid fall in use which occurred between 2001 and 2005 (Fig 2).

The reasons underlying the finding of a stable prevalence of overall MHT use in Australia since about 2005 are unknown, but a component may beongoing use in women with moderate to severe menopausal symptoms. Following publication of the initial results from the WHI trials, regulatory authorities in many countries, including Australia, revised their regulations to recommend the use of MHT for the shortest period of time and at the lowest possible dose, to limit its use to women with moderate or severe symptoms, and to advise against the use of MHT to prevent chronic disease [3,4]. In our study, 9% of all participants in 2013 (73% of current MHT users) reported MHT use for ≥5 years. Similarly, 8% of all participants in the National Health Survey in 2004–5 aged 45–64 years (59% of current users of the same age) reported use for ≥5 years. By contrast, the National Health Survey in 2001 found that 17% of all participants aged 50–69 years (63% of current users in that survey) reported use of MHT for ≥5 years [9]. Although these studies cannot be directly compared due to some differences in age range and methods, collectively the data suggest that the prevalence of long-duration use of MHT decreased substantially, almost halving, from 2001 to 2005, but has remained at broadly similar levels from 2005 to 2013 (Fig 2). The lower absolute proportion of current long duration (≥5 years) users in 2013 compared to that in 2001 suggests more targeted use of MHT. However, our finding that three-quarters of all users have been using MHT for 5 years or more, which corresponds to 9% of all women in their fifties and sixties using MHT for longer durations, is important because MHT-related risks, particularly the risk of developing breast cancer, increase with increasing duration of use [4,12]. Although the decision to use MHT is made by each woman in consultation with her practitioner, and we were not able in the context of a survey such as this to assess the risk/benefit assessments occurring at an individual level which underpin our results, these findings do emphasise the continued need for vigilance in relation to MHT use, particularly long term use.

For women with an intact uterus, systemic oestrogen-only MHT was the second most used type (20%) after combination MHT (39%). In women with a uterus, oestrogen-progestagen formulations have been predominantly used instead of oestrogen-only MHT because of the protective effect of progestagen against endometrial hyperplasia and cancer. An independent review of the worldwide evidence on MHT from trials and observational studies [4], reported a relative risk of breast cancer in current versus never-users of oestrogen-only MHT of 1.2 (95%CI 1.1–1.4) after 5 years and 1.3 (95%CI 1.2–1.5) after 10 years of use in comparison to 1.6 (95%CI 1.5–1.7) and 2.2 (95%CI 2.0–2.4) respectively, for oestrogen-progestagen MHT. Use of combination MHT is associated with greater relative risks of breast and ovarian cancer than oestrogen-only MHT, but does not increase the risk of endometrial cancer for women with a uterus [20]. However, it has been estimated that for women with an intact uterus, the combined risk of breast, endometrial and ovarian cancer after around 5 years of oestrogen-progestagen MHT is greater than that associated with 5 years of oestrogen-only MHT [21,22].

Although it contains largely similar chemical constituents, BHT is not subject to the same regulatory controls as MHT and safety issues have been raised regarding its use, [15,23]. We found that the prevalence of current use of BHT was 2% and ever use was 6%. The latter is consistent with the findings of a study from South Australia [16] which reported that BHT accounted for 6.9% of products ever used for menopausal symptoms, although the number of women who identified what therapy (MHT, BHT, herbal or other) they were taking was small (n = 111). Reports of BHT use outside Australia often originate from the US. A recent study used two on-line surveys to access BHT among women [24]. In the first survey (n = 801), 2% of women aged 45–60 years old reported use of compounded BHT and in the second survey (n = 1771) 21% of women aged 40 years or older reported ever use of BHT. For both surveys, however, participants were invited from an opt-in panel of survey takers instead of a random population-based sample and therefore findings cannot be generalised to the US population. In another US study, 20% of respondents (n = 184) reported being ever users of BHT and 14% were current users [25]. Women surveyed in this US study were attending a women’s health clinic and menopausal centre where prevalence of BHT use is likely to be higher than the general population. Therefore, to our knowledge, our study is one of the first studies to assess BHT prevalence at a national level in any country using a population-based sample.

The current study has a number of strengths, including its relatively large sample size, the random selection of women invited to participate, the use of validated MHT-related questions and collection of data across all Australian states. A number of limitations should be borne in mind when interpreting the findings. First, the participation rate for completed questionnaires was 22% and therefore the observed prevalence rates may not be generalisable to all Australian women. However, there was a broad agreement in relation to several demographic characteristics between LADY study participants and the general female Australian population in the specified age groups, indicating reasonable representativeness of the study population. A second limitation is that we used self-reported data on MHT and BHT use. It should be noted, however, that data from the Pharmaceutical Benefits Scheme (a government program providing subsidised prescription drugs) were unsuitable for estimating overall use of MHT because several formerly commonly used combined MHT preparations are no longer included and because Pharmaceutical Benefits Scheme data on MHT use is based on prescription numbers rather than the number of users. Likewise, MHT utilisation data from the Australian Statistics on Medicine report [26] were also unsuitable because they are based on prescription numbers obtained by combining Pharmaceutical Benefits Scheme data with survey data from a sample of community pharmacies, and therefore figures are not the total number of all MHT prescriptions issued in Australia. In regard to BHT, use can only be assessed from self-reported data because prescriptions are not captured by routinely collected databases.

We found that the population-weighted estimate of women with hysterectomy in the LADY study was 26% and the age-specific estimates were 19%, 23%, 31% and 35% for women aged 50–54, 55–59, 60–64 and 65–69 years, respectively. These findings are consistent with data from the 2004–5 National Health Survey which indicate that 23% of women aged 45–64 years and 33% of women aged ≥65 years reported having a hysterectomy [8] and support the representativeness of the cohort in relation to the general population. The proportion of the sampled population with a prior hysterectomy could potentially impact both the overall estimated prevalence of MHT (or BHT) use, and the estimated overall prevalence by MHT type. We found that oestrogen-only MHT was used by the majority of women (77%) who had a prior hysterectomy and that overall, 44% of current users reported using oestrogen-only MHT. Data from the 2011 Australian Statistics on Medicine report [26] also show systemic oestrogen-only preparations accounting for the majority of MHT prescriptions and estimated the proportion of oestrogen-only prescriptions as 35%. Although these two results cannot be directly compared given the differences in methods, timing and sampling frames, this broad consistency with our finding that oestrogen-only MHT is the most commonly used type is reassuring.

In conclusion, results from the current study interpreted in relation to prior studies, indicate that overall use of MHT in women in their fifties and sixties in Australia has largely stabilised at 12–15% over the last decade, following the substantial drop in MHT use reported from 2001 to 2005. However, almost three-quarters of current users reported use of MHT for ≥5 years, indicating prolonged exposure and consequent health risks. These findings provide a new benchmark of MHT use in Australia; because of the complex safety profile of MHT, continued monitoring of its use within the population seems warranted.
